# Pericardial effusion requiring intervention in patients undergoing percutaneous left atrial appendage occlusion: Prevalence, predictors, and associated in-hospital adverse events from 17,700 procedures in the United States

**DOI:** 10.1016/j.hrthm.2021.05.017

**Published:** 2021-05-18

**Authors:** Muhammad Bilal Munir, Muhammad Zia Khan, Douglas Darden, Deepak Kumar Pasupula, Sudarshan Balla, Frederick T. Han, Ryan Reeves, Jonathan C. Hsu

**Affiliations:** * Section of Electrophysiology, Division of Cardiology, University of California San Diego, La Jolla, California; † Division of Cardiovascular Medicine, West Virginia University Heart & Vascular Institute, Morgantown, West Virginia; ‡ Division of Cardiology, Heart and Vascular Institute, University of Pittsburgh Medical Center, Pittsburgh, Pennsylvania

**Keywords:** Complications, Mortality, National estimates, Pericardial effusion, Watchman

## Abstract

**BACKGROUND:**

Left atrial appendage occlusion has shown promise in mitigating the risk of stroke in selected patients with atrial fibrillation.

**OBJECTIVE:**

The purpose of this study was to determine the real-world prevalence and in-hospital outcomes in left atrial appendage occlusion (Watchman) recipients complicated by pericardial effusion requiring percutaneous drainage or open cardiac surgery–based intervention.

**METHODS:**

Data were derived from the National Inpatient Sample database from January 2015 to December 2017. The primary outcomes assessed were the prevalence of pericardial effusion requiring intervention and in-hospital outcomes including mortality, other major complications, hospital stay > 1 day, and hospitalization costs. Predictors of pericardial effusion requiring intervention were also analyzed.

**RESULTS:**

Pericardial effusion requiring intervention occurred in 220 total patients (1.24%). After multivariable adjustment, pericardial effusion requiring intervention was associated with in-hospital mortality (adjusted odds ratio [aOR] 511.6; 95% confidence interval [CI] 122–2145.3), other Watchman-related major complications (aOR 1.35; 95% CI 0.83–2.19), length of stay > 1 day (aOR 17.64; 95% CI 12.56–24.77), and hospitalization cost above the median of $24,327 (aOR 3.58; 95% CI 2.61–4.91). Independent patient predictors of pericardial effusion requiring intervention from the procedure included advanced age (aOR 1.029 per 1-year increase; 95% CI 1.009–1.05 per 1-year increase), higher CHA_2_DS_2_VASc score (aOR 1.221 per 1-point increase; 95% CI 1.083–1.377 per 1-point increase), and obesity (aOR 2.033; 95% CI 1.464–2.823).

**CONCLUSION:**

In a large, contemporary real-world cohort of Watchman recipients in US practice, the prevalence of pericardial effusion requiring intervention was 1.24%. Pericardial effusion requiring intervention was associated with several adverse events including increased in-hospital mortality, other major complications, prolonged hospital stay, and hospitalization costs.

## Introduction

Atrial fibrillation (AF) is an important cause of cardioembolic stroke as it results in thrombus formation in the left atrial appendage (LAA) in >90% of patients.^1^ Although coumadin and direct oral anticoagulants are effective in reducing AF-associated stroke risk, their use is limited by patient compliance and adverse effects.^2–4^ LAA occlusion using a Watchman device (Boston Scientific, MA) has shown promising results as an alternative to stroke risk reduction in selected patients with AF. The landmark PROTECT AF (percutaneous closure of the left atrial appendage vs warfarin therapy for prevention of stroke in patients with atrial fibrillation) trial showed Watchman implantation to be noninferior to coumadin with respect to the primary composite end point of stroke, systemic embolism, and cardiovascular death.^5^ However, a periprocedural safety hazard was identified in this trial as close to 5% of patients sustained serious pericardial effusion requiring percutaneous drainage or open cardiac surgery–based intervention. Subsequent studies such as PREVAIL (prospective randomized evaluation of the Watchman left atrial appendage closure device in patients with atrial fibrillation vs long-term warfarin therapy) trial and CAP (continued access to PROTECT AF) registry showed reduction in rates for serious pericardial effusion.^6,7^

As one of the most feared complications from an LAA occlusion procedure, pericardial effusion requiring intervention from LAA occlusion is assumed to be associated with significant morbidity and mortality; however, this association and its magnitude have not been studied in a large population of Watchman recipients. There are also limited real-world data on the prevalence of pericardial effusion requiring percutaneous drainage or open cardiac surgery–based intervention in patients implanted with a Watchman device outside of controlled clinical trials. Furthermore, patient-level predictors of pericardial effusion requiring intervention have not been identified and may provide insight into who may be most at risk. We aimed to study these parameters from a nationally representative US population sample.

## Methods

For the purpose of the present analysis, data were derived from the National Inpatient Sample (NIS) for calendar years 2015–2017. The year 2015 was taken as a starting year for our analysis since the Watchman device was approved by the Food and Drug Administration in that year. The NIS is made possible by a Federal-state-industry partnership sponsored by the Agency for Healthcare Research and Quality. The NIS is derived from all states for national estimates of health care utilization, costs, and outcomes. NIS data are compiled annually, and therefore data can be used for analyses of disease trends over time. The NIS approximates 20% of all discharges from all US non-Federal hospitals and provides discharge weights that are used for the computation of national estimates.^8^ Institutional review board approval and informed consents were not required for this study given the de-identified nature of the NIS data set and public availability. The NIS adheres to the 2013 Declaration of Helsinki for conduction of human research.

We identified patients undergoing Watchman implantation using *International Classification of Diseases, Ninth Revision, Clinical Modification* (*ICD-9-CM*) and *International Classification of Diseases, Tenth Revision, Clinical Modification* (*ICD-10-CM*) codes of 37.90 and 02L73DK, respectively. Patients younger than 18 years were excluded. The study population was then divided into patients who sustained pericardial effusion requiring percutaneous drainage or open cardiac surgery–based intervention and those patients who did not have this complication. For percutaneous drainage, the *ICD-9-CM* code of 37.0 and *ICD-10CM* codes of 0W9D30Z and 0W9D3ZZ were used. For the open cardiac surgery–based intervention, the *ICD-9-CM* code of 37.1 and *ICD-10-CM* codes of 0W9D00Z and 0W9D0ZZ were used. Baseline characteristics, in-hospital outcomes, and key complications were compared in Watchman recipients on the basis of the presence or absence of pericardial effusion requiring percutaneous drainage or open cardiac surgery–based intervention. For computing hospitalization costs, the cost-to-charge ratio files supplied by the Healthcare Cost and Utilization Project were applied to the total hospital charges and adjusted for inflation to December 2017. Independent associations of pericardial effusion requiring intervention (vs not) with in-hospital mortality, *major complications* (defined as composite of cardiac arrest, myocardial infarction, ischemic stroke, hemorrhagic stroke, transient ischemic attack, major bleeding, and vascular complication), length of stay > 1 day, and hospitalization cost above the median of $24,327 were analyzed. Additionally, the independent associations of pericardial effusion requiring open cardiac surgery–based intervention vs percutaneous drainage with in-hospital mortality, major complications, length of stay > 1 day, and hospitalization cost above the median of $24,327 were analyzed. We also assessed patient-level predictors of pericardial effusion requiring intervention in our study cohort.

Descriptive statistics are presented as frequency with percentage for categorical variables and as median with interquartile range (IQR) for continuous variables. Baseline characteristics were compared using the Pearson *X*^2^ test and Fisher exact test for categorical variables and the Mann-Whitney *U* test for continuous variables. Logistic regression was performed to estimate odds ratios (ORs) with 95% confidence intervals (CIs) to determine patient-level predictors of pericardial effusion requiring percutaneous drainage or open cardiac surgery–based intervention. A forward stepwise entry model was used for this purpose. Initially, all variables, which were significantly associated with pericardial effusion with a *P* value of <.05 in univariable analysis, were entered into the model from the baseline table. Subsequently, only those variables were retained in the model that were associated with pericardial effusion with a *P* value of <.10 during forward entry. For the assessment of the independent association of pericardial effusion requiring intervention with key outcomes including in-hospital mortality, other major complication, length of stay > 1 day, and hospitalization cost above the median of $24,327, a single-step multivariable-adjusted regression model was used. Age, sex, race/ethnicity, CHA_2_DS_2_-VASc score, hospital bed size, and standard comorbidities were used to adjust the model. A type I error rate of <.05 was considered statistically significant. All statistical analyses were performed using SPSS version 26 (IBM Corporation, Armonk, NY) and R version 3.6. Because of the complex survey design of the NIS, sample weights, strata, and clusters were applied to the raw data to generate national estimates.

## Results

From January 2015 to December 2017, a total of 17,700 patients were implanted with a Watchman LAA occlusion device. Pericardial effusion requiring percutaneous drainage or open cardiac surgery–based intervention occurred in 220 total patients (1.24%). The baseline characteristics of the study population stratified on the basis of pericardial effusion requiring intervention (vs not) are summarized in Table 1. The prevalence of pericardial effusion requiring intervention was more in female patients undergoing Watchman implantation (61.4% vs 39.8%; *P* < .001). Patients with a CHA_2_DS_2_-VASc score of 5 and 6 or more were generally more prone to the development of pericardial effusion requiring intervention (18.2% vs 12.6%; *P* < .001 and 7.8% vs 4.9%; *P* < 0.001, respectively). Comorbidities such as deficiency anemia (18.2% vs 13.8%; *P* < .001), coagulopathy (9.1% vs 4.8%; *P* <.001), congestive heart failure (34.1% vs 32.4%; *P* < .001), obesity (22.7% vs 15.2%; *P* < .001), and complicated diabetes (15.9% vs 12.3%; *P* < .001) were more common in patients with pericardial effusion requiring intervention.

In-hospital outcomes and important Watchman-related complications stratified on the basis of pericardial effusion requiring intervention (vs not) are presented in Tables 2 and 3, respectively. Death was more common in patients with pericardial effusion requiring intervention than in those who did not have this complication (11.4% vs 0.1%; *P* < .001). Patients with pericardial effusion requiring intervention had a higher prevalence of other Watchman-related complications than did patients without pericardial effusion requiring intervention (38.6% vs 9.3%; *P* <.001). This is primarily composed of any other cardiovascular (25% vs 2.1%; *P* < .001) and pulmonary (20.5% vs 2.9%; *P* < .001) complications, respectively. Nonhome discharges were more prevalent in the pericardial effusion requiring intervention cohort (15.4% vs 3.3%; *P* < .001). Patients with pericardial effusion requiring intervention were also noted to have a longer median length of stay (4 days [IQR 2–6 days] vs 1 day [IQR 1–1 day]; *P* < .001) and an increased total cost of hospitalization ($36,767$ [IQR $27,846–$50,411] vs $24,275 [IQR $18,607–$30,072]; *P* < .001).

**Figure 1:**
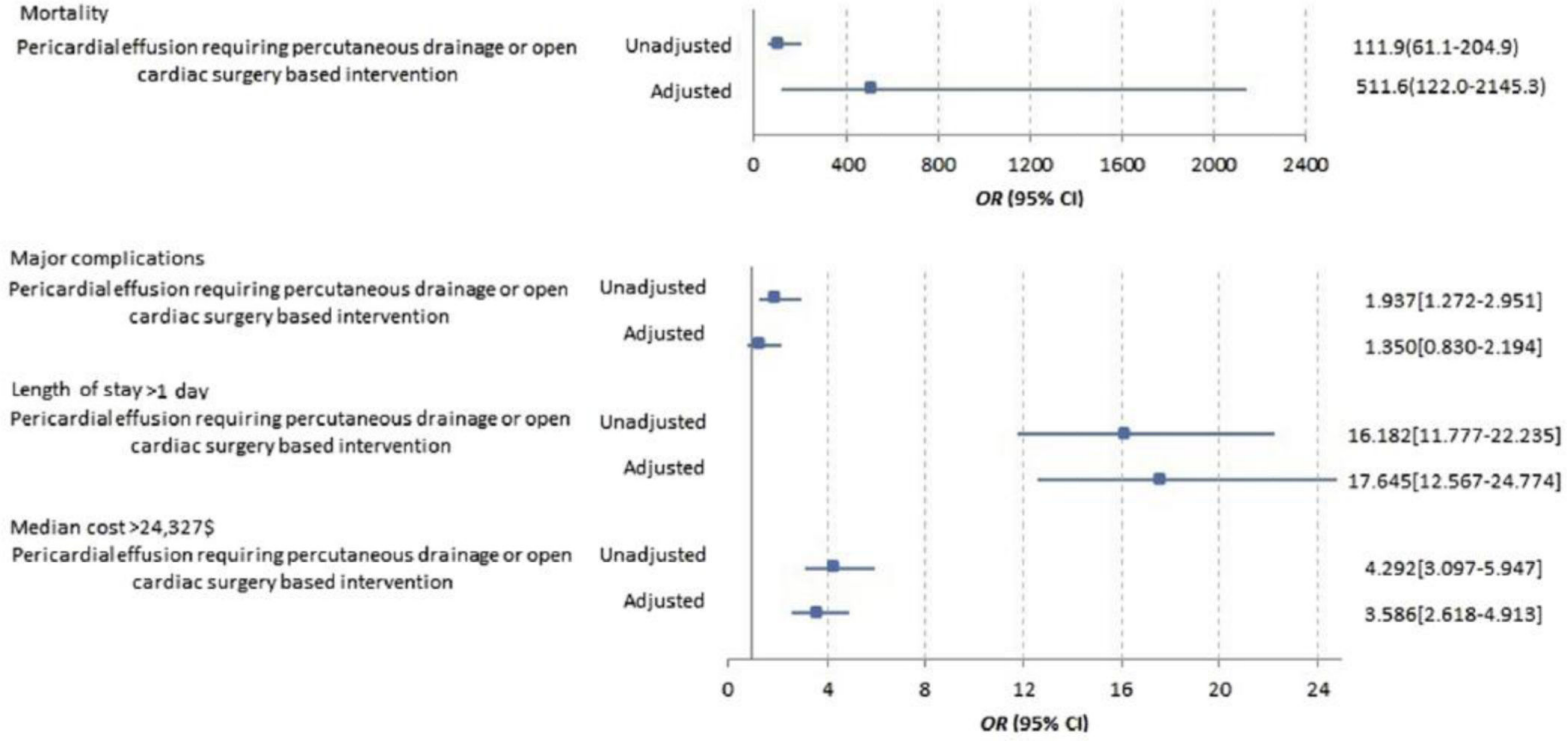
Adjusted association of pericardial effusion requiring intervention with inpatient mortality, other major Watchman-related complications, prolonged length of stay, and increased hospitalization costs. CI = confidence interval; OR = odds ratio. Adjusted jfor Age, Sex, Race, CHA_2_DS_2_-VASc, hospital bedsize and selected comorbid conditions

To assess the independent association of the complication of pericardial effusion requiring intervention with other in-hospital outcomes, we constructed multivariable models adjusting for potential confounders. After adjustment, pericardial effusion requiring intervention was independently associated with increased mortality (adjusted OR [aOR] 511.6; 95% CI 122–2145.3), increased rate of other Watchman-related major complications (aOR 1.35; 95% CI 0.83–2.19), length of stay > 1 day (aOR 17.64; 95% CI 12.56–24.77), and hospitalization cost higher than the median of $24,327 (aOR 3.58; 95% CI 2.61–4.91). Please see Figure 1 for detailed results. The independent associations of pericardial effusion requiring open cardiac surgery– based intervention vs percutaneous drainage with outcomes of in-hospital mortality, major complications, length of stay > 1 day, and hospitalization cost above the median of $24,327 are shown in Online Supplemental Figure 1. No significant differences in in-hospital mortality (aOR 2.00; 95% CI 0.25–15.67), other Watchman-related major complications (aOR 0.63; 95% CI 0.14–2.76), and length of stay > 1 day (aOR 0.62; 95% CI 0.18–2.10) were observed in patients who underwent open cardiac surgery–based intervention vs percutaneous drainage of pericardial effusion.

**Figure 2:**
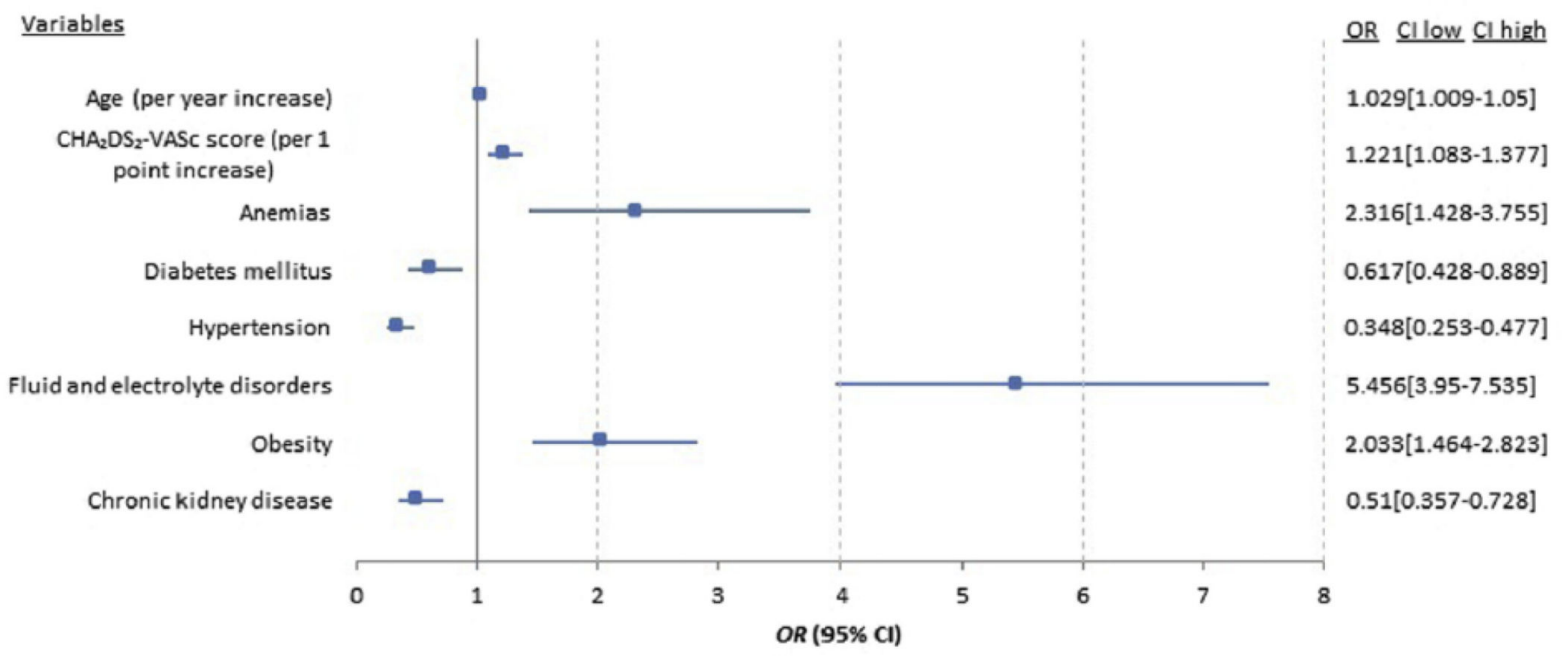
Patient-level predictors of pericardial effusion requiring intervention. CI = confidence interval; OR = odds ratio. Age, Sex, Race, CHA_2_DS_2_-VASc hospital bedsize and selected comorbid conditions used to derive a final model with forward entry (p<0.1)

Patient-level characteristics that predicted pericardial effusion requiring intervention are shown in Figure 2. After multivariable adjustment, advanced age (OR 1.029 per 1-year increase; 95% CI 1.009–1.05 per 1-year increase), higher CHA_2_DS_2_-VASc score (OR 1.221 per 1-point increase; 95% CI 1.083–1.377 per 1-point increase), anemia (OR 2.316; 95% CI 1.428–3.755), and obesity (OR 2.033; 95% CI 1.464–2.823) were associated with a higher odds of pericardial effusion requiring intervention. Diabetes (OR 0.617; 95% CI 0.428–0.889), hypertension (OR 0.348; 95% CI 0.253–0.477), and chronic kidney disease (OR 0.51; 95% CI 0.357–0.728) were associated with a lower odds of pericardial effusion requiring intervention.

## Discussion

The main findings of the present investigation are as follows: (1) In a large, contemporary, real-world cohort of patients undergoing Watchman LAA occlusion implantation, the overall prevalence of pericardial effusion requiring percutaneous drainage or open cardiac surgery–based intervention was 1.24%.^2^ Patients undergoing Watchman implantation complicated by pericardial effusion requiring intervention (vs those who did not have this complication) had higher mortality, other Watchman-related complications, hospital length of >1 day, and increased hospitalization costs.^3^ Patient-level characteristics that predicted those who were at increased risk of having pericardial effusion requiring intervention included advanced age, higher CHA_2_DS_2_-VASc score, and obesity whereas a history of diabetes, hypertension, and chronic kidney disease was associated with a less risk of having pericardial effusion requiring intervention.

Mechanical LAA occlusion using a Watchman device has shown promise in reducing stroke risk associated with AF, especially in patients who are intolerant to oral anticoagulants.^9,10^ The landmark PROTECT AF trial demonstrated benefit of Watchman implantation with respect to stroke risk reduction in patients with AF but also showed an increased rate of periprocedural pericardial effusion requiring percutaneous drainage or open cardiac surgery–based intervention.^5^ The overall rate of serious pericardial effusion requiring intervention in the PROTECT AF trial was close to 5%.^5^ Furthermore, the incidence of pericardial effusion was significantly more in the first half of the PROTECT AF trial (6.3%) as compared with the second half of the study (3.7%). The complimentary registry to the PROTECT AF trial (CAP registry) showed a reduced incidence of pericardial effusion requiring intervention with a reported rate of 2.2%.^7^ The incidence of pericardial effusion requiring intervention further lowered to w2% in the PREVAIL trial.^6^ In a more recent study from the NCDR Left Atrial Appendage Occlusion Registry, the incidence of pericardial effusion requiring intervention was reported to be w1.39%.^11^ Our real-world analysis of >17,000 Watchman recipients showed the prevalence of pericardial effusion requiring intervention to be w1.24%, which is similar to the reported data from the NCDR Left Atrial Appendage Occlusion Registry. With the clinical availability of a new generation Watchman FLX device, the rate of pericardial effusion requiring intervention in real-world clinical practice is expected to reduce further. The Watchman FLX device was analyzed in the pivotal Protection Against Embolism for Nonvalvular AF Patients: Investigational Device Evaluation of the Watchman FLX LAA Closure Technology trial and showed no incidence of pericardial effusion requiring intervention at 7 days postimplantation.^12^

Because of a significant number of pericardial effusions requiring intervention experienced in our cohort, it is worthwhile to assess the possible mechanisms leading to this complication during Watchman implantation. Earlier studies have demonstrated that nearly all procedural aspects of Watchman implantation have been associated with a risk of significant pericardial effusion. In a root cause analysis from the CAP registry,^7^ which encompassed a thorough review of procedural details, most pericardial effusions were related to the placement of adjunctive devices such as guidewires or catheters in the LAA (18%) and actual deployment of the Watchman device itself (18%). This was then followed by delivery system manipulation within the LAA (14%) and transseptal puncture (9%) in descending order. Increased familiarity and experience with various procedural steps involved in Watchman implantation by the operators will continue to mitigate this risk of significant pericardial effusion and subsequent mortality. Our study has highlighted certain patient-related factors such as advanced age, higher CHA_2_DS_2_-VASc score, and obesity that are associated with an increased risk of pericardial effusion requiring intervention and that can guide implanting physicians in risk stratification before the procedure. On the contrary, we have also shown that the presence of other comorbidities such as diabetes, hypertension, and chronic kidney disease were associated with a lower risk of pericardial effusion requiring intervention. The exact mechanism why these comorbidities were associated with a lower risk of significant pericardial effusion is unknown but should be the subject of future studies. The new generation Watchman FLX device has a lower profile design and a predeployment ball with protected distal struts and may be more safely manipulated in the LAA before deployment, thereby reducing the risk of traumatic perforation and resulting pericardial effusion^12^; however, this has yet to be examined in large real-world cohorts using the new generation device.

Our study also showed increased resource utilization in patients with pericardial effusion after Watchman implantation. This is expected, as all patients needed either percutaneous drainage or open cardiac surgery–based intervention of such effusions that subsequently prolonged hospital length of stay as well as associated costs. Further reduction in the rate of clinically significant pericardial effusion will have the potential to make Watchman devices more cost effective.

### Limitations

Our study has following key limitations: (1) The NIS is an administrative claims–based database that uses *ICD* codes, which may be prone to errors. The hard clinical end points such as mortality and pericardial effusion, however, are less subject to error. Additionally, Agency for Healthcare Research and Quality quality control measures are routinely instituted that guarantee data integrity.^8^ Furthermore, the *ICD-9* code used in this study was not specific to the Watchman device and could be referred for any LAA occlusion procedure. Because of the limited magnitude of other research study of endocardial devices and any epicardial LAA occlusion procedures performed in the United States, we believe that the application of this code for the purpose of our study was able to mostly characterize Watchman implants.^13^ (2) The NIS captures only inpatient admissions and does not provide any information on outpatient encounters. This limitation may result in selection bias; however, our data are well representative of the national utilization of Watchman devices performed during inpatient settings; in fact, since inpatient hospitalization is often required for reimbursement for the procedure, our results may be more indicative of widespread practice.^14^ (3) The NIS censors data gathering at discharge, so long-term outcomes could not be ascertained from the present data set. (4) Specific data on potential confounders including medications, LAA morphology, and operator and intraprocedural characteristics could not be examined from the NIS.^5^ The NIS is also limited by the lack of more granular data on imaging modalities used to guide LAA occlusion and also does not contain information on the likely causative intraprocedural etiologies for pericardial effusion in the setting of LAA occlusion.

## Conclusion

In this large real-world registry of Watchman recipients, we found the prevalence of pericardial effusion requiring percutaneous drainage or open cardiac surgery–based intervention to be 1.24%. Pericardial effusion requiring intervention was independently associated with inpatient mortality, other major Watchman-related complications, prolonged length of stay, and increased hospitalization costs.

## Supplementary Material

Supplement 1

## Figures and Tables

**Table 1:** Baseline characteristics of the study population of Watchman recipients stratified on the basis of having pericardial effusion requiring intervention vs not

Variable	Pericardial effusion requiring intervention (n = 220)	No pericardial effusion requiring intervention (n = 17,480)	*P*
Age (y)	78.5 (72.5–82.75)	76 (71–82)	<.001
Female sex	135 (61.4)	6,960 (39.8)	<.001
Race/ethnicity			
White	185 (84.1)	14,465 (86.1)	<.001
Black	<10 (<2.3)	690 (4.1)	
Hispanics	<10 (<2.3)	925 (5.5)	
Others	25 (11.4)	725 (4.3)	
CHA_2_DS_2_-VASc score			
2	35 (15.9)	2,740 (15.7)	<.001
3	50 (22.7)	5,730 (32.8)	
4	70 (31.8)	5,240 (30)	
5	40 (18.2)	2,200 (12.6)	
≥6	15 (7.8)	860 (4.9)	
Median score	4 (3–4.75)	3 (3–4)	.016
Comorbidities			
Deficiency anemia	40 (18.2)	2,410 (13.8)	<.001
Congestive heart failure	75 (34.1)	5,665 (32.4)	<.001
Chronic pulmonary disease	40 (18.2)	3,715 (21.3)	<.001
Coagulopathy	20 (9.1)	840 (4.8)	<.001
Cerebrovascular disease	<10 (<4)	1,380 (7.9)	<.001
Diabetes	30 (13.6)	3,745 (21.4)	<.001
Diabetes with complications	35 (15.9)	2,145 (12.3)	<.001
Hypertension	115 (52.3)	11,165 (63.9)	<.001
Alcohol abuse	40 (18.1)	791 (4.5)	<.001
Liver disease	<10 (<2.3)	430 (2.5)	<.001
Obesity	50 (22.7)	2,655 (15.2)	<.001
Peripheral vascular disorders	30 (13.6)	1,940 (11.1)	<.001
Chronic kidney disease	35 (15.9)	3,775 (21.6)	<.001
Valvular disease	0 (0.0)	45 (0.3)	<.001
Hospital location			
Rural	0 (0.0)	215 (1.2)	<.001
Urban nonteaching	20 (9.1)	1,750 (10.0)	
Urban teaching	200 (90.9)	15,515 (88.8)	
Bed size			
Small	25 (11.4)	1,890 (10.8)	<.001
Medium	60 (27.3)	3,830 (21.9)	
Large	135 (61.4)	11,760 (67.3)	
Census division			
New England	0 (0.0)	540 (3.1)	<.001
Mid-Atlantic	35 (15.9)	2,250 (12.9)	
East North Central	30 (13.6)	2,410 (13.8)	
West North Central	<10 (<2.3)	1,180 (6.8)	
South Atlantic	50 (22.7)	3,790 (21.7)	
East South Central	<10 (<4)	810 (4.6)	
West South Central	40 (18.2)	2,055 (11.8)	
Mountain	20 (9.1)	1,680 (9.6)	
Pacific	30 (13.6)	2,765 (15.8)	
Payee			
Medicare/Medicaid	205 (93.2)	15,750 (90.4)	<.001
Private insurance	<10 (<4)	1,425 (8.2)	
Self-pay	0 (0.0)	65 (0.4)	
Other	<10 (<2.3)	185 (1.1)	
Median income			
0th–25th	50 (23.3)	3,480 (20.2)	<.001
26th–50th	50 (23.3)	4,210 (24.5)	
51th–75th	40 (18.6)	4,930 (28.7)	
76th–100th	75 (34.9)	4,575 (26.6)	

Values are presented as median (interquartile range) or n (%).

For N < 10, the absolute numbers are not reported as per Healthcare Cost and Utilization Project recommendations.

**Table 2:** Hospital outcomes and resource utilization in Watchman recipients

Outcome	Pericardial effusion requiring intervention (n = 220)	No pericardial effusion requiring intervention (n = 17,480)	*P*
Died at discharge	25 (11.4)	20 (0.1)	<.001
Discharge disposition			
Home/routine/self-care	165 (84.6)	16,880 (96.7)	<.001
Nonhome discharges	30 (15.4)	580 (3.3)	
Resource utilization			
Length of stay (d)	4 (2–6)	1 (1–1)	<.001
Cost of hospitalization ($)	36,767 (27,846–50,411)	24,275 (18,607–30,702)	<.001

Values are presented as median (interquartile range) or n (%).

**Table 3:** Complications in patients undergoing Watchman implantation stratified on the basis of having pericardial effusion requiring intervention vs not

Variable	Pericardial effusion requiring intervention (n = 220)	No pericardial effusion requiring intervention (n = 17,480)	*P*
Other complications	85 (38.6)	1625 (9.3)	<.001
Major complications*	25 (11.4)	1085 (6.2)	<.001
Cardiovascular complications			
Any cardiovascular complication	55 (25.0)	365 (2.1)	<.001
Percutaneous coronary intervention	<10 (<4)	25 (0.1)	<.001
Cardiac arrest	<10 (<4)	<10 (<0.2)	<.001
Heart block	0 (0.0)	190 (1.1)	.180
ST-segment elevation myocardial infarction	<10 (<2.3)	20 (0.1)	<.001
Non–ST-segment elevation myocardial infarction	<10 (<2.3)	60 (0.3)	.001
Cardiogenic shock	25 (11.4)	40 (0.2)	<.001
Systemic complications			
Any systemic complication	0 (0.0)	40 (0.2)	>.99
Anaphylaxis	0 (0.0)	<10 (0.0)	>.99
Arterial embolism	0 (0.0)	25 (0.1)	>.99
Septic shock	0 (0.0)	<10 (0.1)	>.99
Vascular complications			
Any peripheral vascular complication	<10 (<2.3)	190 (1.1)	.094
AV fistula	0 (0.0)	25 (0.1)	>.99
Pseudoaneurysm	0 (0.0)	60 (0.3)	>.99
Access site hematoma	0 (0.0)	40 (0.2)	>.99
Retroperitoneal bleeding	0 (0.0)	30 (0.2)	>.99
Venous thromboembolism	<10 (<2.3)	45 (0.3)	<.001
Neurological complications			
Any neurological complication	0 (0.0)	250 (1.4)	.080
Hemorrhagic stroke	0 (0.0)	70 (0.4)	>.99
Ischemic stroke	0 (0.0)	140 (0.8)	.425
Transient ischemic attack	0 (0.0)	40 (0.2)	>.99
Pulmonary complications			
Any pulmonary complications	45 (20.5)	500 (2.9)	<.001
Respiratory failure	25 (11.4)	295 (1.7)	<.001
Pneumothorax	15 (6.8)	80 (0.5)	<.001
Pleural effusion	0 (0.0)	90 (0.5)	.632
Pneumonia	<10 (<4)	75 (0.4)	<.001
Need for a prolonged ventilator (>36 h)	20 (9.1)	135 (0.8)	<.001

Values are presented as n (%).

For N < 10, the absolute numbers are not reported as per Healthcare Cost and Utilization Project recommendations.

AV = atrioventricular.

* Defined as composite of cardiac arrest, myocardial infarction, ischemic stroke, hemorrhagic stroke, transient ischemic attack, major bleeding, and vascular complication.
