# Supplementary material for: Pericardial effusion requiring intervention in patients undergoing percutaneous left atrial appendage occlusion: Prevalence, predictors, and associated in-hospital adverse events from 17,700 procedures in the United States
Source: Heart Rhythm. Author manuscript; Available in PMC 2022 Sep 1. (PMC8558825; doi:10.1016/j.hrthm.2021.05.017)
Supplement: Supplement 1 [file NIHMS1732568-supplement-Supplement_1.pdf]

Supplemental Figure:

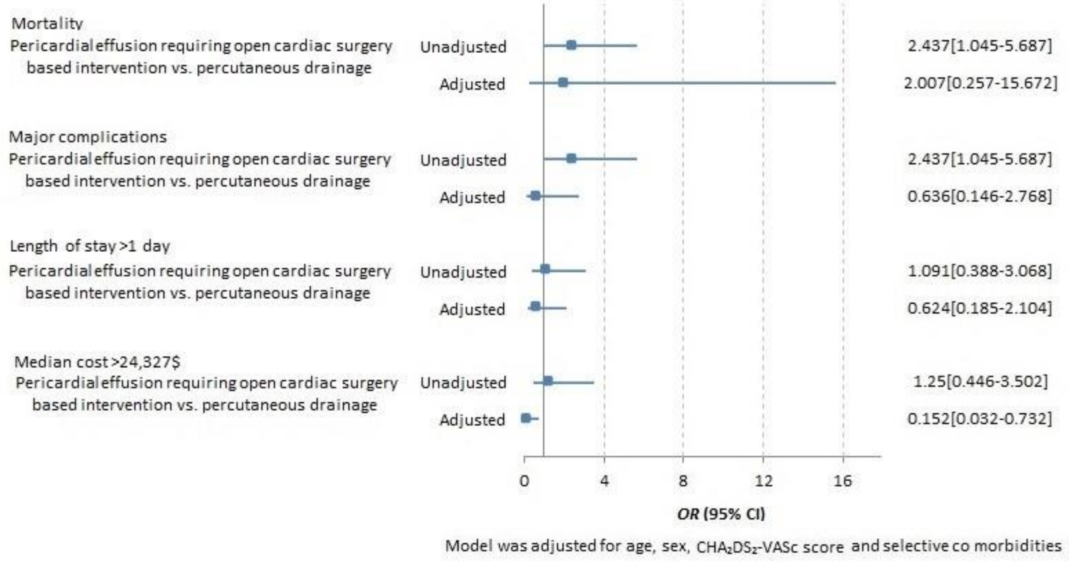

Adjusted association of pericardial effusion requiring open cardiac surgery based intervention versus percutaneous drainage with in-patient mortality, other major Watchman related complications, prolonged length of stay and increased hospitalization costs
